# The role of immunotherapy in microsatellites stable metastatic colorectal cancer: state of the art and future perspectives

**DOI:** 10.3389/fonc.2023.1161048

**Published:** 2023-05-03

**Authors:** Annalice Gandini, Silvia Puglisi, Chiara Pirrone, Valentino Martelli, Fabio Catalano, Simone Nardin, Andreas Seeber, Alberto Puccini, Stefania Sciallero

**Affiliations:** ^1^ Medical Oncology Unit 1, IRCCS - Ospedale Policlinico San Martino, Genoa, Italy; ^2^ Department of Haematology and Oncology, Medical University of Innsbruck, Innsbruck, Austria; ^3^ IRCCS Humanitas Research Hospital, Humanitas Cancer Center, Medical Oncology and Haematology Unit, Rozzano, Milan, Italy

**Keywords:** microsatellite stable, MSS, colorectal cancer, immunotherapy, checkpoint inhibitors, combination strategy, immunomodulation, microbiome

## Abstract

Colorectal cancer (CRC) is the third leading cause of cancer-related deaths worldwide, despite several advances has been achieved in last decades. Few prognostic and predictive biomarkers guide therapeutic choice in metastatic CRC (mCRC), among which DNA mismatch repair deficiency and/or microsatellite instability (dMMR/MSI) holds a crucial role. Tumors characterized by dMMR/MSI benefit from immune checkpoint inhibitors. However, most of the mCRC patients (around 95%) are microsatellite stable (MSS), thereby intrinsically resistant to immunotherapy. This represents a clear unmet need for more effective treatments in this population of patients. In this review, we aim to analyze immune-resistance mechanisms and therapeutic strategies to overcome them, such as combinations of immunotherapy and chemotherapy, radiotherapy or target therapies specifically in MSS mCRC. We also explored both available and potential biomarkers that may better select MSS mCRC patients for immunotherapy. Lastly, we provide a brief overview on future perspectives in this field, such as the gut microbiome and its potential role as immunomodulator.

## 1 Introduction

Colorectal cancer (CRC) is the third leading cause of cancer-related deaths in men and in women, and its global incidence is continuously increasing (1). During the last two decades, a tremendous improvement in outcome has been achieved in metastatic CRC (mCRC), mainly due to the introduction of novel drugs and biomarker-driven patient selection.

However, to date, only a few biomarkers have sufficient actionable and clinical implications to guide treatment choice, such as RAS and BRAF mutations and DNA mismatch repair deficiency and/or microsatellite instability (dMMR/MSI). Indeed, CRC patients harboring dMMR/MSI tumor showed outstanding and practice-changing results with immune checkpoints inhibitors, doubling progression free survival (PFS) and maintaining durable response (2). Unfortunately, only 15% of early stages CRC and 5% of mCRC are dMMR/MSI (3), thus the vast majority of mCRC patients do not benefit from this treatment approach. Because of this, scientific community is focusing on better understanding mechanisms behind the intrinsic resistance to immunotherapy in order to overcome them. This field is a big unmet need in CRC, and many trials have been carried on: in this review we would like to walk through the available data on immunotherapy, alone or in combination, in pMMR/MSS metastatic CRCs to better understand how far along we are and what the main gaps are.

## 2 Understanding immuno-resistance in microsatellites stable colorectal cancers

CRC carcinogenesis and immunity are a complex system that derives from interactions on different levels. The majority (85%) of CRC presents a chromosomal instability and are typically MSS (4), while 15% have genetic instability, resulting in high MSI status. Finally, epigenetic is driven by hypermethylation or hypomethylation and modulates the expression of certain genes without any genetic alterations (5). About microsatellite instability, it is related to defects in the mismatch repair system which lead to mutations across the genome and, consequently, cause the release of many mutation-associated neoantigens (MANA) that enhance the immunity response: this is why dMMR/MSI tumors are highly responsive to immunotherapeutic agents (6). Indeed, tumor mutational burden (TMB) is directly proportional to the production of neoantigens and, consequently, can induce a pro-inflammatory tumor microenvironment (TME) (5, 7). Since CRCs with a proficient mismatch-repair pathway do not accumulate mutations, TMB is low and TME is not pro-inflammatory, defining them as “cold” tumors. Due to that, several studies were conducted to find a way to enhance the production of neoantigens, although therapeutic implications of this approach are still under investigation (7).

In addition to the primarily low involvement of the immune system, immunity cells may be selected to limit their efficiency according to the so called “immune exclusion” phenomenon (8). Indeed, preclinical experiments demonstrated different T-cell populations inside deficient (dMMR) and proficient-MMR (pMMR) tumors, in terms of both cell quantity and heterogeneity (9): it seems that in pMMR/MSS tumors immunity cells react against the tumor, but their presence is localized all around it, without reaching the core; thus compromising efficacy of immunotherapy. In this regard, preclinical models investigated the role of transforming growth factor-beta (TGF-b), which prevents T cell tumor infiltration through induction of fibrosis (10). Moreover, the up-regulation of oncogenic pathways, like WNT/beta-catenin signaling pathway and MAP-kinase pathway, has shown to contribute to immune-exclusion trough silencing the activation of T-cells (11). In particular, about 60% of MSS CRCs have an up-regulation of the MAP-kinase pathway, which leads to a reduction of Major Histocompatibility Complex (MHC) class I molecules’ expression and a decrease of the number of CD8-positive T cells in the tumor core (12). Also, some immunosuppressive patterns seem to be involved in poor responses to immunotherapy. PIK3CA mutations, that can be found in about 13% of CRCs regardless the MMR status (13) and can be secondary to a loss of Phosphatase and tensin homolog (PTEN) (14), are associated with Programmed Death-Ligand 1 (PDL-1) expression and immunosuppressive effect.

In addition, both intensity and quality of the TME can influence response to immunotherapy (15): a high prevalence of FOXP3 positive regulatory T cells (Treg) or myeloid derived suppressor cells (MDSC) is associated with poor prognosis in several neoplasms, although their role in CRCs is more complex and under further investigation (6, 16).

Furthermore, it is important to underline the role of the Vascular Endothelial Growth Factor (VEGF), that can induce endothelial cell proliferation, thus promoting neo-angiogenesis in the tumor mass (17). Along with the mechanisms whereby the tumor cells evade immunity system, the adaptation to a hypoxic environment represents a powerful stimulus for up-regulating genes involved in proliferation, glycolysis and angiogenesis, often related to aggressive and metastatic tumor behavior (18). VEGF is also a mediator of immunosuppression by promoting the accumulation of MDSCs and Tregs (19) thus enhancing its potential involvement in immune exclusion.

Such complex mechanisms contribute to create a “cold” tumor environment and could represent potential targets for novel therapeutic strategies to overcome resistance to immunotherapy, even if specific targets and biomarkers need further investigation.

## 3 Therapeutic strategies to overcome immunotherapy resistance

To overcome resistance and enhance an effective immune response against tumor cells, several trials are investigating immunotherapy-based combination strategies to synergistically stimulate the tumor microenvironment in order to promote immune cell recruitment in MSS mCRC (20).

### 3.1 Immunotherapy plus chemotherapy

Based on the rational that chemotherapy may interrupt mechanisms of immune tolerance and, consequently, enhance cancer cells’ immunogenicity (21), several trials were designed to study the association of chemotherapy and immunotherapy (**Table 1**).

**Table 1:** Immunotherapy plus Chemotherapy.

**STUDY** **(#=non cited in the text)** **REF**	**AGENTS**	**PHASE**	**SETTING**	**%MSS**	**PRIMARY ENDPOINTS**	**RESULTS** * *In the experimental arm when applicable
**NCT03832621** **MAYA trial** (22)	nivolumab + ipilimumab + temozolomide	II	Metastatic or inoperable MSS -MGMT silenced, I line	100	8 months PFS rate	36%
**NCT03519412**	temozolomide + pembrolizumab	II	Metastatic, I line	100	ORR	Ongoing
**NCT03721653** **ATEZOTRIBE** (23)	FOLFOXIRI + bevacizumab + atezolizumab	II	Unresectable, I line	94	PFS	13.1 months
**NCT03414983** **Checkmate9x8** (24)	mFOLFOX + bevacizumab ± nivolumab	II	Unresectable metastatic, I line	NA	PFS	11.9 months
**NCT02860546** (25)	trifluridine/tipiracil + nivolumab	II	Metastatic or locally advanced, II line and beyond	100	irORR	0%
**NCT05229003#**	anlotinib + irinotecan and anlotinib + penpulimab + irinotecan	II	Recurrent/metastatic, II line and beyond	NA	ORR	Ongoing
**NCT04866862#**	fruquintinib + camrelizumab	II	Metastatic, III line	NA	ORR	Ongoing
**NCT04262687#**	capecitabine + oxaliplatin + bevacizumab + pembrolizumab	III	Metastatic, I line	NA	10 months OS	Ongoing

NA, not available.

In preclinical models, acquired resistance to the clinical agent temozolomide (TMZ) could inactivate the MMR system and, therefore, increase the mutational burden and trigger immune surveillance (26). Based on this result, the MAYA trial evaluated the efficacy and safety of an immune-sensitizing strategy through an induction therapy with temozolomide followed by a combination of low-dose ipilimumab and nivolumab in patients with MSS and MGMT-silenced mCRCs. Median progression free survival (mPFS) was 7 months and median overall survival (mOS) 18.4 months, with objective response rate (ORR) of 45%. These data provide a “proof of concept” that induction therapy with temozolomide followed by immunotherapy could induce sustained clinical benefit with a good tolerability (22). The applicability in the clinical practice needs further investigations since the selection of patients could represent an important limit: among the 716 patients prescreened, only 135 started the first part of the treatment and, among these, only 24% of patients could start the second part. Similar results are expected by the ARETHUSA trial, which selects patients based on MMR status, MGMT expression for the induction with TMZ and, at the end, TMB for treatment with pembrolizumab: this trial is still recruiting and data are immature [NCT03519412].

In first line, positive results came from the ATEZOTRIBE trial, where patients with previously untreated pMMR/MSS mCRC were randomized to receive mFOLFOXIRI (5fluoro-uracil, leucovorin, oxaliplatin and irinotecan) plus bevacizumab with or without atezolizumab. After 19.9 months of follow-up, median PFS was 13.1 months in the atezolizumab group and 11.5 months in the control group (hazard ratio HR=0·69 p=0·012), suggesting that this strategy could give a benefit in this setting, although the benefit was probably restricted to the subgroup of MSI mCRC patients included in the trial (23).

At the American Society of Clinical Oncology (ASCO) Gastrointestinal Cancers Symposium in 2022, Lenz et al. reported the results of Checkmate 9x8, a phase II study which assessed the role of nivolumab added to the standard first line therapy (mFOLFOX plus bevacizumab) in mCRC, regardless of the MMR status. Primary endpoint was PFS and in both arms mPFS was 11.9 months with HR of 0.81, which did not meet the prespecified threshold for statistical significance. However, nivolumab plus standard of care (SOC) showed higher PFS rates at 15 months (45% VS 21.5%) with no difference between MSS and MSI patients, a higher objective response rate (60% vs 46%), and more durable responses (12.9 months vs 9.3 months), with acceptable safety (24).

Moving to the maintenance setting, unsatisfactory results came from the umbrella trial MODUL in which patients were treated with fluoropyrimidine plus bevacizumab alone or with an experimental biomarker-driven treatment based on histopathological characteristics (5FU/LV plus cetuximab and vemurafenib in *BRAF^V600E^* mutated, capecitabine plus trastuzumab and pertuzumab in HER2 positive, fluoropyrimidine plus bevacizumab and atezolizumab or cobimetinib plus atezolizumab in wild-type patients). At the moment, only results on cohort 2 have been published: endpoints of efficacy were not reached in the experimental arm with atezolizumab in MSS mCRC (27, 28).

Several combination trials were performed also in further lines.

Patel at al., in a phase II trial investigated the combination of trifluridine–tipiracil plus nivolumab in heavily pretreated MSS mCRC. The trial was negative with no clinical benefit: none of the patient enrolled achieved objective response and the trial was interrupted (25).

To date, combining immunotherapy and chemotherapy in MSS mCRC patients did not obtain clinically meaningful results thus this strategy is not currently available in clinical practice.

### 3.2 Immunotherapy plus target therapy and tyrosine kinase inhibitors

In preclinical models, the combination of immunotherapy and tyrosine kinase inhibitors (TKI) in pMMR/MSS CRC cells seems to increase the production of neoantigens and, consequently, to induce immune-mediated cell death (29). Therefore, Fukuoka et al. developed the REGONIVO trial [EPOC1603], a phase I trial assessing efficacy of nivolumab plus regorafenib in CRC and gastric cancer. Only one out of the 25 CRC patients was dMMR/MSI. In the results presented, the cohort of patients with CRC showed a median PFS of 7.8 months with one-year PFS rate of 41.7% and one-year OS rate of 68%. Interestingly, patients with lung metastases had higher objective response rates when compared with those with liver metastases (30).

The phase II evaluating the combination regorafenib and nivolumab has just been published (31) and confirms the lack of effect of this combination in patients with liver metastases (mPFS 11.9 months in patients without liver involvement, versus 1.8 months in those with hepatic disease).

Kim et al. evaluated the same combination in a phase I/Ib trial and reached a mPFS of 4.3 months and mOS of 11.1 months (32). Similar results were found with regorafenib in association with pembrolizumab that obtained a median PFS of 2.0 (1.8 -3.5) months and median OS of 10.9 (5.3-not reached) months (33) in heavily pre-treated mCRC patients.

The association of regorafenib with avelumab was evaluated in the single-arm phase II trial REGOMUNE, but it did not show objective response rates, with stable disease as best response in 53.5% of the 48 patients enrolled. Median PFS and median OS were 3.6 and 10.8 months, respectively (34).

Lenvatinib in combination with immunotherapy has demonstrated great efficacy in other malignancies, such as endometrial cancer. LEAP-005 is a phase 2 study assessing ORR and safety of the combination in previously treated solid tumors, including pMMR/MSS CRC. In particular, this subgroup of patients was treated in third line and reached an objective response rate of 22% and a mPFS of 2.3 months, with manageable toxicities (35). The phase III randomized trial is ongoing to investigate this combination in larger cohorts [LEAP-017 NCT04776148].

Slightly better results were obtained from the CAMILLA trial, were pMMR mCRC patients who progressed after 2 or more lines of therapy were treated with durvalumab plus cabozantinib. The trial followed preclinical results in which cabozantinib with anti-PD1 showed to slow tumor’s growth and increase expression of the CD4+ T cell ligand HLA-DR on the tumor cells themselves (36). Despite the different immunotherapeutic agent used, CAMILLA’s efficacy analyses revealed an ORR of almost 30% and a disease control rate of 86.2% (25/29), with a median PFS of 4.4 months and a median OS of 9.1 months (37).

The BEACON trial in 2019 (38) set a new therapeutic paradigm for *BRAF^V600E^* mutated mCRC and preclinical data demonstrated that the combination of BRAF inhibitor plus epidermal growth factor receptor (EGFR) inhibitor could induce a transient MSI phenotype. On this background, Van Morris and Colleagues designed a phase I/II trial with the triplet encorafenib, cetuximab and nivolumab in *BRAF^V600E^* pretreated MSS mCRC. The results presented at ASCO Gastrointestinal Cancers Symposium in 2022 are promising, reaching an ORR of 50%, a median PFS of 7.4 months (vs 4.2 in the BEACON) and a median OS of 15.1 months (vs 9.3 months in the BEACON trial) (39). The randomized phase II trial [NCT05308446] is actually ongoing to confirm these preliminary results.

Several preclinical models and different studies have suggested that MEK inhibition can lead to up-regulation of MHC I and increase the infiltration of CD8+ into the tumors (40, 41).

Therefore, different trials investigating the synergic role of immunotherapy plus MEK inhibitors were developed.

In 2016 Bendell et al. presented the results of a phase Ib trial which assessed the clinical activity of atezolizumab plus cobimetinib in 24 patients with pretreated mCRC. The ORR was 17% and, among responders, three patients with major response were MSS (42). The same combination has been evaluated by Hellman and Colleagues in a cohort of 84 CCRs of which 74% were MSS. They reported a response rate in seven patients (8% of the cohort), of whom six patients had microsatellite stable status (43). IMblaze370 is a multicenter, open-label, phase 3, randomized, controlled trial, that was opened in 73 academic medical centers among 11 countries, whose results were published in 2019 on Lancet Oncology. The 363 patients with pre-treated unresectable or metastatic CRC, mostly with microsatellite stability, were randomized to receive atezolizumab alone or in combination with cobimetinib or SOC (regorafenib). Unfortunately, this trial did not met its primary endpoint of improving overall survival (OS) in the experimental arm compared to SOC, registering a mOS of 8.9 months in the combination arm, 2.1 months in the atezolizumab alone arm and 8.5 months in the regorafenib arm: these results demonstrated lack of benefit using immunotherapy, with or without the combination of a MEK inhibitor, in patients with low levels of inflammation (44). Despite the unsatisfactory results, few more trials are ongoing evaluating cobimetinib in association with nivolumab and ipilimumab [NCT02060188] or atezolizumab and bevacizumab [NCT02876224] in pretreated MSS mCRC.

To date, most of the clinical trials investigating the combination of immunotherapy and TKI in MSS mCRC failed to show clinically significant results, that may be partly explained with poor selection of heavily refractory mCRC MSS patients. In **Table 2** are summarized the main trials available and ongoing. These negative results highlight the strong need for a better understanding of underlying mechanisms of immune-resistance and synergistic effects between different drugs.

**Table 2:** Immunotherapy plus target therapy and Tyrosine kinase inhibitors.

STUDY (#=non cited in the text)	AGENTS	PHASE	SETTING	%MSS	PRIMARY ENDPOINT	RESULTS* *In the experimental arm when applicable
**NCT03406871** **REGONIVO TRIAL** (30)	regorafenib + nivolumab	Ib	Advanced or metastatic, >II line	96	Dose limiting toxicity (DLT)	regorafenib 80mg/die
**NCT03712943**	regorafenib + nivolumab	I/Ib	Advanced or metastatic, >I line	100	Maximum tolerated dose (MTD) and DLT	regorafenib 80mg/die
**NCT04126733** (31)	regorafenib + nivolumab	II	Advanced or metastatic, II or III line	100	ORR	7%
**NCT03657641** (33)	regorafenib + pembrolizumab	I/II	Advanced or metastatic, II /IV line	100	mPFS	2.0 months
**NCT03475953** **REGOMUNE** (34)	regorafenib + avelumab	II	Advanced non resectable/ metastatic, pretreated	100	ORR	Ongoing
**NCT03797326** **LEAP-005** (35)	lenvatinib + pembrolizumab Colorectal cancer cohort	II	Metastatic and/or unresectable, pretreated >2 line	100	ORR	22%
**NCT04776148** **LEAP-017**	lenvatinib + pembrolizumab	III	Advanced	NA	PFS	Ongoing
**NCT03539822** **CAMILLA TRIAL** (37)	durvalumab + cabozantinib	I/II	Metastatic, III line and beyond	100	ORR	30%
**NCT04017650** **SWOG S2107** (39)	encorafenib + cetuximab + nivolumab	I/II	Metastatic, II line and beyond	100	ORR, Safety	45%
**NCT01988896** (42)	atezolizumab + cobimetinib Colorectal cancer cohort	I/Ib	Metastatic any line	74	ORR	8%
**NCT02788279** **IMBlaze370** (34)	atezolizumab + cobimetinib vs regorafenib	III	Locally advanced or metastatic, III line	95	OS	8.87 months
**NCT02876224#**	cobimetinib + aezolizumab + bevacizumab	Ib	Unresectable metastatic, II line and beyond	NA	Safety	Ongoing
**NCT03642067#**	nivolumab + relatlimab	II	Metastatic, II line and beyond	NA	ORR, PR, CR	Ongoing
**NCT04110093#**	regorafenib + anti PD-1 (nivolumab, canrelizumab, sintilimab, toripalimab)	II	Metastatic, III line and beyond	NA	ORR, PFS	Ongoing
**NCT05382741#**	durvalumab + regorafenib	IIb	Adjuvant (IV stage NED)	NA	DFS	Ongoing
**NCT05409417#**	tislellimab + CAPOX + bevacizumab tislelizumab + mFOLFOX + cetuximab	II/III	Metastatic (liver metastasis)	NA	conversion rate, safety	Ongoing
**NCT04963283#**	cabozantinib + nivolumab	II	Metastatic or unresectable, II line	NA	DCR	Ongoing
**NCT03608046#**	avelumab + cetuximab + irinotecan	II	Metastatic, II line	NA	ORR	Ongoing

NA, not available.

### 3.3 Immunotherapy plus antiangiogenetic or anti-EGFR agents

The anti-angiogenic bevacizumab seems to potentiate dendritic cells’ functions, to facilitate CD8+ lymphocytes infiltration into tumor and to decrease Tregs functions, important studies such as the MODUL trial, cited above, and the BACCI trial (capecitabine in association with bevacizumab and atezolizumab in refractory mCRC) (45) were then developed, but both reported negative results in all the subgroups. Other studies which aim to assess the role of bevacizumab in association with immunotherapy [NCT03396926, NCT02848443] are reported in **Table 3**, but results are still unknown. Moreover, in a humanized mice model, the association of cobimetinib, bevacizumab and pembrolizumab was not able to act on tumor growth. However, an immune modulation in TILs was observed, suggesting that the combination could potentially enhance immune susceptibility in MSS CRC (36). The use of dual therapy with VEGF inhibitors and immunotherapy has been widely evaluated in many different malignancies, although no thrilling results were obtained in CRCs (24, 45, 49, 50).

**Table 3:** Immunotherapy plus antiangiogenetic.

STUDY (#=non cited in the text)	AGENT	PHASE	SETTING	%MSS	PRIMARY ENDPOINT	RESULTS^*^ *In the experimental arm when applicable
**NCT03442569** (46)	nivolumab + ipilimumab + panitumumab	II	Unresectable or metastatic, II and beyond	100	12 weeks ORR	35%
**NCT03174405** **AVETUX TRIAL** (47)	avelumab + cetuximab + FOLFOX	II	Metastatic, I line	93	PFS rate at 12 months	NA ORR 79.5%
**NCT04561336** **CAVE TRIAL** (48)	avelumab + cetuximab	II	Preatreted, metastatic	92	OS	11.6 months
**NCT04513951** **AVETRIC TRIAL**	avelumab + cetuximab + FOLFOXIRI	II	Unresectable, I line	NA	ORR	Ongoing
**NCT03396926#**	pembrolizumab + capecitabine + bevacizumab	II	Unresectable, metastatic, II line and beyond	100	ORR	5%
**NCT02873195#**	capecitabine + bevacizumab + atezolizumab	II	Metastatic, II line and beyond	83	PFS	4.4 months
**NCT05314101#**	tislelizumab + bevacizumb + trifluridine/tipiracil	II	Metastatic (liver metastasis), III and beyond	NA	PFS	Ongoing
**NCT04194359#**	sintilimab + CAPOX + bevacizumab	II/III	Metastatic, I line	NA	PFS	Ongoing

NA, not available.

Preclinical data showed that anti-EGFR therapy can contribute to activate a tumor-specific adaptive immune response and immunogenic apoptosis, often associated with an increased expression of CTLA-4 and PD-L1, during the development of treatment resistance (51). Therefore, several attempts have been made to evaluate association of anti-EGFR and immunotherapy (**Table 3**).

Preliminary results of a trial evaluating if the addition of ipilimumab and nivolumab to panitumumab would increase response rate in patients with *RAS/BRAF* wild-type MSS mCRC were published last year. Among 49 enrolled patients, the 12-week response rate was 35% with median PFS of 5.7 months, meeting the prespecified primary endpoint (46).

Afterwards, Stein and colleagues developed the AVETUX trial, a single arm trial that combined in first line mFOLFOX6 and avelumab with cetuximab in *RAS/BRAF* wild-type mCRC patients. The primary endpoint of 12 months PFS rate was not reached, but high tumor responses were observed, especially in terms of depth of response (47).

On the same basis, Martinelli et al. reported results from the randomized phase II CAVE trial to evaluate the efficacy of rechallenge with cetuximab in association with avelumab in the third-line in RAS wild-type mCRC with no selection regarding microsatellite status: the trial showed a promising median OS of 11.6 months (48), suggesting potential synergism between immune checkpoint inhibitors and anti-EGFR drugs.

Moreover, cetuximab and avelumab were also evaluated in first line in association with mFOLFOXIRI in the AVETRIC trial: it has just closed the enrolment and results are not available yet [NCT04513951].

### 3.4 Combination of immune checkpoint inhibitors

Combined blockade with immunotherapy strategies has been explored to overcome immune resistance (**Table 4**).

**Table 4:** Combination of immune checkpoint inhibitors.

STUDY (#=non cited in the text)	AGENTS	PHASE	SETTING	%MSS	PRIMARY ENDPONT	RESULTS^*^ *In the experimental arm when applicable
**NCT02870920** (52)	durvalumab + tremelimumab VS best supportive care	II	Metastatic, pretreated	99	OS	6.6 vs 4.1 months
**NCT02720068** (53)	pembrolizumab + favelizumab	I	Metastatic, third or more	100	safety	Manageable, antitumor activity observed
**NCT03860272** (54)	botensilimab ± balstilimab	I/II	Metastatic, pretreated	100	ORR, DCR	24%, 73%
**NCT03377361** **CheckMate 9N9**	nivolumab + trametinib ± ipilimumab	I/II	Metastatic, pretreated	100	Safety and tolerability	Ongoing
**NCT03642067#**	nivolumab + relatlimab	II	Metastatic or locally advanced	NA	ORR	Ongoing

NA, not available.

The NCT02870920 study evaluated the combination of durvalumab (anti PDL1) and tremelimumab (anti- Cytotoxic T-Lymphocyte Antigen 4 CTLA-4) versus best supportive care for refractory mCRC. Disease control rate (DCR) was of 22.6% and 6.6%, median PFS was not prolonged (1.8 months vs. 1.9 months), but median OS was longer in the experimental group (6.6 months vs 4.1 months). It is important to notice that patients with a TMB > 28 MTs/MB benefited more from dual immunotherapy, whereas high TMB in the best supportive care group was associated with a poor prognosis, enhancing the relative benefit (52).

NCT03860272 is the first trial of botensilimab, a novel innate/adaptive immune activator against CTLA-4, in association with the anti PD1 balstilimab in patients with advanced cancer. Patients were heavily pretreated, including 14/34 treated with prior immunotherapy, and received botensilimab at 1 or 2 mg/kg every 6 weeks plus balstilimab 3 mg/kg every 2 weeks. The ORR was 24% (10/41), with a DCR of 73% (30/41) (54).

The combination of nivolumab and trametinib with or without ipilimumab in previously treated cancer of the colon or rectum is being tested in an ongoing phase I/II trial [CheckMate 9N9-NCT03377361].

Interesting results, even if still immature, came from the use of antibodies against the lymphocyte activation gene-3 (LAG3). LAG-3 is a surface molecule expressed by immunity cells that plays a role in the regulation of lymphocytes and dendritic cells’ activity (55). It showed a potential role in cancer treatment in both preclinical and clinical studies, since its inhibition could potentially trigger an inflammatory phenotype (56).

The combination of pembrolizumab and the anti-LAG3 antibody favezelimab in previously treated MSS mCRC patients [NCT02720068], showed a median OS of 8.3 months and a median PFS of 2.1 months in a phase I study. Of 89 patients receiving the combined blockade, 4 patients achieved a partial response and 1 showed a complete response. Median duration of response was 10.6 months (range 5.6–12.7) (53).

Although the association of immune-checkpoint inhibitors blockade has not shown to be as effective as in dMMR/MSI counterpart, novel combinations demonstrated promising activity also in MSS mCRC, particularly among well-selected patients without liver metastases. Thus large phase III trials are ongoing and results are eagerly awaited.

### 3.5 Immunotherapy in combination with radiotherapy

Radiotherapy induces tumor-cell death and increases the expression of MHC class I on cell membrane, improving antigen presentation by dendritic cells with a strong immune activation (57). Some evidence showed, moreover, that it can also induce the so-called “abscopal effect”, a rare phenomenon that consists of tumor regression in a site distant from the field of irradiation due to the activation of immune system against cancer cells. Therefore, it was suggested that combining radiotherapy with immunotherapy could represent a good strategy for enhancing the immune system reaction (58). By now, the combination of RT plus immunotherapy has been studied especially in non-small cell lung cancer (NSCLC) and melanoma, because of their different background in tumor immunogenic biology (59). Unfortunately, in CRC, and especially in MSS CRC, only a few data are available (**Table 5**). A recent phase 2 trial [NCT03104439] has evaluated the combination of fractioned radiotherapy (8 Gy in three fractions to a single metastatic lesion) with ipilimumab and nivolumab in 40 patients with MSS metastatic colorectal and pancreatic cancer. Disease control rate in the intention to treat population was 25% with ORR of 10%; median PFS was 2.4 months, and median OS 7.1 months. However, 13 out of 40 patients did not receive radiotherapy: among the 27 patients treated with the protocol-defined radiotherapy, responses were higher, with a DCR of 37% and an ORR of 15%. The median duration of disease control was more than 15 months. It is important to notice, though, that half of the patients had grade 3-4 adverse events (60). Segal’s phase II trial failed at its primary endpoint of ORR in non-irradiated lesions but in rare instances a systemic immune augmentation and a regression at these sites was observed, supporting abscopal response with a manageable safety profile (61).

**Table 5:** Combination of immunotherapy plus radiotherapy.

STUDY (#=non cited in the text)	AGENTS	PHASE	SETTING	%MSS	PRIMARY ENDPOINT	RESULTS* *In the experimental arm when applicable
**NCT03104439** (60)	radiotherapy 8Gy in 3Fx + nivolumab + ipilimumab	II	Metastatic first line	100	DCR, ORR	25%, 10%
**NCT03122509**	radiotherapy + durvalumab + tremelimumab	II	Metastatic second line	100	ORR in not irradiated lesions	8.3% (95% CI, 1.0% to 27%)
**NCT02888743**	radiotherapy on liver + durvalumab + tremelimumab	II	Metastatic >1 line	NA	Safety and tolerability	Ongoing
**NCT03122509**	radiotherapy standard/ablation + durvalumab + tremelimumab	II	Metastatic >2 line	100	Efficacy and safety, ORR	Ongoing
**NCT03007407**	durvalumab + tremelimumab after radiation therapy	II	Metastatic	100	ORR	Ongoing
**NCT04108481**	(Y90-RE) + durvalumab	I/II	Metastatic	100	Safety	Ongoing
**NCT02992912**	atezolizumab and stereotactic ablative radiotherapy (SABR 45Gy in 3Fx)	II	Metastatic	NA	PFS	Ongoing
**NCT03507699**	nivolumab + ipilimumab + CMP-001 (a TLR9 agonist) and radiosurgery	I	Metastatic >1 line	100	Safety and tolerability	Ongoing
**NCT02437071#**	pembrolizumab + Radiation (A) or Ablation (B)	II	Metastatic, third or more	NA	ORR	Interim A: 9%

NA, not available.

Moreover, several trials on the combination of durvalumab and tremelimumab plus radiotherapy are ongoing and no data are available. Here we report few examples to underline the interest of the scientific community in this promising field:

-NCT02888743 is a randomized phase II trial designed to investigate the safety of durvalumab and tremelimumab with or without high or low-dose radiation therapy in patients with metastatic colorectal or non-small cell lung cancer.
-NCT03122509 is evaluating the efficacy and safety of durvalumab and tremelimumab plus radiotherapy in metastatic CRC patients who are undergoing to radiotherapy as standard therapy or plus ablation.
-NCT03007407 evaluates the safety and response to the combination of durvalumab plus tremelimumab when given after radiation therapy for patients with MSS mCRC.
-NCT04108481 is a single-centre, open-label, Phase I/II trial that evaluates the feasibility and safety of Yttrium-90 radioembolization (Y90-RE) in combination with durvalumab 750 mg in subjects with liver-predominant MSS mCRC.

About other immune checkpoint inhibitors, the results of a phase II study with a combination of atezolizumab and radiotherapy in pretreated MSS mCRC [NCT02992912] and of a combination study with nivolumab, ipilimumab, CMP-001 (a TLR9 agonist) and radiosurgery (21Gy in 3 fractions) in patients with mCRC and liver metastases [NCT03507699], are still not available.

## 4 Biomarkers for patients’ selection

The majority of efforts in pMMR/MSS mCRCs treated with immune checkpoint inhibitors have been focused on predictive factors of response to such therapies: several biomarkers have been evaluated in the last years, investigating the tumoral micro-environment, the mutational landscape, the immune system, and the clinical characteristics of the patients.

Despite all these attempts, it is not possible to draw clear conclusions in favour of one biomarker or another. In this part of the review, we will summarize the most promising results and the still existing areas of uncertainty.

The PD-1 receptor and its ligand PD-L1 expression are two well-known biomarkers; however the actual prevalence of PD-L1 expression in mCRC is not completely clear. Moreover, the few available data on these are limited to dMMR/MSI CRC, and they did not show strong positive results (62–64).

### 4.1 Tumor mutational burden

High TMB levels can be detected in about 3% of MSS mCRC. Therefore, the prevalence of TMB-high could be more significant than expected in the mCRC population (65), implying the necessity of testing it on a large scale (66). However, it is difficult to define when a tumor is TMB-high and when it is low, since several methods of analysis and ways to express the results are available (67). Moreover, the optimal threshold is far from being clearly assessed: Schrock et al. reported a cut-off of 37-41 mut/Mb (68), in the REGONIVO trial the cut-off was 22.5 mut/Mb [REGONIVO JCO], while in CCTG CO.26 trial it was 28 mut/Mb (52). The basket trial TAPUR evaluated the efficacy of already available target agents in metastatic solid tumors showing specific somatic genomic variants (69). For the TMB-high pMMR/MSS mCRC cohort, the *a priori* cut-off was > 9 mut/Mb, and the patients were allocated to receive different immune checkpoint inhibitors. Overall, 27 patients have been enrolled in the pembrolizumab subgroup: 7 showed benefit from the treatment for six months or more, and two out of the three patients treated for at least one year showed a TMB > 40 mut/Mb. The mPFS was 9.3 weeks, the mOS 51.9 weeks, and the 1-year OS rate 45.6%; moreover, the DCR was 28% and the ORR was 11% (70).

In the nivolumab plus ipilimumab arm, the median TMB was 13, ranging from 9 to 233 mut/Mb. This subgroup showed lower DC rate (10%) and mOS (42.9 weeks) and a higher toxicity. Given these results, the Authors concluded that the regimen should not be further investigated in TMB-high pMMR/MSS mCRC population, preferring to focus on other treatment strategies (71).

Despite these not-so-encouraging results, there still may be a place for high TMB as a predictive biomarker with immune double blockage regimens, as shown in the CCTG CO.26 trial. Here, heavily pre-treated mCRC patients received durvalumab plus tremelimumab versus the best supportive care: the greatest OS benefit was found for the pMMR/MSS mCRC with plasma TMB higher than 28 mut/Mb (HR 0.34; 90% CI, 0.18 – 0.63; P = .004) (52).

### 4.2 POLE/POLD1 mutations

According to the Cancer Genome Atlas results, one-quarter of hypermutated CRC presents DNA Polymerase Exonuclease Domain (*POLE/POLD1*) mutations (72), that are typically linked to a high TMB. In the pMMR/MSS population, POLE mutations have been identified in about 3% of the cases (73).

Wang et al. analysed a data set of different solid tumors treated with immune checkpoint inhibitors: the frequencies of *POLE* and *POLD1* mutations were 2.79% and 1.37%, respectively, with a high prevalence in CRC (circa 7%); in the overall population, 74% of the *POLE/POLD1* mutated patients also had a pMMR/MSS phenotype. In this work, mutations of *POLE* and *POLD1* were demonstrated to be positive predictive factors of response to immunotherapy since the OS was 34 months, more than doubled when compared with the non-mutated counterpart (74). This positive result has also been confirmed by the multivariable Cox regression analysis, which showed *POLE/POLD1* as an independent biomarker for ICI response.

This higher response rate is probably linked to the high presence of cytotoxic T cells (especially CD8^+^ lymphocytes) and effector cytokines in the tumor microenvironment (75). Nevertheless, more robust preclinical and clinical evidence is needed to assess the predictive role of *POLE/POLD1* mutations in MSS mCRC (76).

### 4.3 The consensus molecular subtypes

Understanding the biology underlying MSS colorectal tumors will lead to improved clinical trial design and to the identification of clinical biomarkers relevant to this population. Because of this, efforts were made in the last years to organize the heterogeneous molecular landscapes of CRC, leading to the Consensus Molecular Subtypes (CMS) classification by the Colorectal Cancer Subtyping Consortium (77). This categorization consists of four different subgroups of tumors: the CMS1 (“immune subtype”, about 14% of all cases) characterized by dMMR/MSI phenotype, hypermutated status, *BRAF^V600E^* mutation; the CMS2 (“canonical”, about 37%) with chromosomal instability, MYC, Wnt and EGFR pathway activation; the CMS3 (“metabolic”, 13%), showing epithelial features and mutations in MAPK pathway; the CMS4 (“mesenchymal”, 23%) with constitutively activated VEGFR pathway. The remaining 13% of the patients shows mixed features, defining a transition phenotype.

The CMS1 and CMS4 subgroups are characterized by strong immune infiltration in their microenvironment, defining them as “hot” tumors with intense immune activation. However, the molecular features and cellular subpopulations differ: CMS1 shows an immunoreactive environment, with T-helper 1, CTLA4, and IDO1 overexpression. In contrast, CMS4 shows an immunosuppressive landscape, mainly characterized by M2 macrophages, Th17 cells, and TGF-b overexpression (78).

Above all, TGF-b raised interest in the last years as a potential target of specific drugs. Recently, a phase II trial evaluated the anti-PD-L1 antibody/TGF-b trap bintrafusp alfa combined with radiation therapy of metastatic lesions in CMS4 mCRC: unfortunately, no patients had any benefit in terms of disease response (ORR 0%) or survival (mPFS 1.6 months; mOS 5 months) (79); therefore, further efforts have to be done in this population.

Lenz et al. first evaluated CMS predictive role on a large population in the CHECKMATE 9X8 trial (24), which randomized MSS mCRC patients to receive nivolumab or placebo in combination with first-line FOLFOX plus bevacizumab. The trial was negative; however, in the exploratory analyses, the anti-PD-1 regimen showed a clinical benefit in CMS1 and CMS3 subgroups, where almost one patient out of three was free from progression at 20 months. This finding supports the idea of the possible usefulness of CMS as a predictive biomarker for immune checkpoint inhibitors.

## 5 Interpretation and future perspectives

As previously described, the majority of CRCs are characterized by microsatellites stability and so, due to the clinical relevance, several attempts of “immunizing” cold tumors have been made. Unfortunately, it is still unclear what the best strategy might be, and we would like to underline through this review which ones seem to be more promising and deserve to be carried on.

Unfortunately, among all the combination strategies, some of them have shown unacceptable toxicities or absence of response (durvalumab plus tremelimumab) (80).

On the other hand, the association of regorafenib with nivolumab probably leads to the development of a synergic effect that enhances the response and allow to overcome resistance to ICI (30). Despite several limits, such as the absence of synergic effect in presence of liver metastasis, the modest survival benefit and the absence of predictive biomarkers, this treatment could represent a valid therapeutic option for patients affected by CRC without liver metastasis (ORR 22% vs 0%) (81).

In addition, data of the combination of the novel second-generation CTLA4-inhibitor botensilimab plus the PD-1 inhibitor balstilimab give a hint of enhanced efficacy, due to the DCR of 96% in patients without liver metastasis, and no severe toxicity (54).

Finally, the association of pembrolizumab and the anti-LAG3 favezelimab in pretreated MSS mCRC seems promising, and data from the ongoing phase III trial are expected.

Research is also active on developing cancer vaccines, based on cancer-specific neoepitopes bounded by T-cells in order to enhance immunological response (82). In the last few years, different type of vaccines has been developed from cancer cell or vector-based, but with progress on next generation sequencing techniques, nano-vaccines and neoantigen vaccines are lastly prevailing (83). Nowadays they are considered a possible strategy to overcome the resistance of MSS mCRC to immunotherapy. Several trials are ongoing (**Table 6**) but data are still immature.

**Table 6:** Cancer vaccine.

STUDY (#=non cited in the text)	AGENTS	PHASE	SETTING	%MSS	PRIMARY ENDPOINT	RESULTS* *In the experimental arm when applicable
**NCT03313778#**	mRNA 4157 ± pembrolizumab	I	Locally advanced or metastatic	NA	Safety, immune response	Ongoing
**NCT03948763#**	mRNA 5671 ± pembrolizumab	I	Locally advanced or metastatic	NA	Safety, immune response, ORR	Ongoing
**NCT04117087#**	KRAS peptide vaccine + nivolumab + ipilimumab	I	Metastatic	NA	Safety, immune response	Ongoing
**NCT04799431#**	Neoantigen Vaccine with Poly-ICLC + retifanlimab	I	Metastatic	NA	Safety	Ongoing
**NCT03639714#**	GRT-C901, GRT- R902 + ipilimumab + nivolumab	I/II	Locally advanced or metastatic	NA	Safety, ORR	Ongoing
**NCT03953235#**	GRT-C903, GRT- R904, ipilimumab + nivolumab	I/II	Locally advanced or metastatic	NA	Safety, ORR	Ongoing
**NCT04912765#**	Neoantigen Dendritic Cell Vaccine + nivolumab	II	Liver metastasis from CRC	NA	24 months RFS, immune response	Ongoing
**NCT05243862#**	PolyPEPI1018, Montanide™ ISA51VG + atezolizumab	II	Metastatic	NA	Safety	Ongoing
**NCT05141721#**	GRT-C901, GRT- R902 + ipilimumab	II/III	Metastatic >1 line	NA	ctDNA response, PFS	Ongoing

MSS, Microsatellite stable; MGMT, O-6-Methylguanine-DNA Methyltransferase; PFS, Progression free survival; mPFS, Median progression free survival; irORR, Immune-Related Overall Response Rate; MPR, major pathological response rate; ORR, overall response rate; OS, overall survival; mOS, Median overall survival; RD, Recommended dose; MTD, Maximum Tolerated Dose; G3, Grade 3; PR, Partial response; CR, Complete response; DCR, Disease Control Rate; FOLFOX, leucovorin calcium (folinic acid), fluorouracil, oxaliplatin; FOLFOXIRI, leucovorin calcium (folinic acid), fluorouracil, and oxaliplatin, irinotecan; CAPOX, Capecitabine plus oxaliplatin; LAG 3, Lymphocyte activation gene ;  TLR9, toll like receptor 9l mRNA, Messenger RNA; RFS, Relapse-free survival; SOC, standard of care; ctDNA, Circulating tumor DNA; NA, not available.

Regarding biomarkers, the significant predictive value of response to ICI of harbouring a pathogenic variant of *POLE/POLD* (84) needs to be deepened since it is still unclear which *POLE* mutations must be determined. A promising possibility seems to be combining *POLE* mutation and TMB, in order to identify patients that could benefit most from immunotherapy.

Moreover, the clinical presentation of pMMR/MSS mCRC is becoming relevant as a reliable predictive factor and in particular the presence of liver metastases, as shown in the REGONIVO trial (30). Indeed, In the mCRC subgroup (25 patients), ORR was 33.3% with significant differences according to metastatic sites. Indeed, the ORR was 63.3% in the patients with lung or nodal metastatic disease and 8.3% in the patients with liver involvement. These results are consistent with those observed in other advanced tumors (85) and suggest that the liver micro-environment may show immunosuppressive features (86), with low tumor control by immune checkpoint inhibitors (87).

As a new avenue, recent evidence uncovered the central role that intestinal microbiome could play.

The human gut contains a huge variety of species and metabolites that can interact with other tissues, modulating their functioning (88). Moreover, it has been demonstrated that the prevalence of different bacteria species could also modify the risk of developing cancer, especially through the activation of inflammatory pathways including NF-kB, IL-6, TNF and other cytokines (89). For example*, E. Coli, C. Jejuni* and *F. nucleatum* produce metabolites that can induce oncogenic changes (89). Based on this knowledge, lot of studies have been made in order to evaluate if microbiota could be used to modulate prevention and therapy. Indeed, previously studies have demonstrated that some of the gut microbioma’s species can contribute not only to prevent or enhance the risk of CRC, but, furthermore, to modulate efficacy and toxicity of some chemotherapeutic drugs (89, 90).

A study conducted on patients affected by gastrointestinal cancers, for whom faecal samplings before and after chemotherapy were collected, showed, a modification of the pattern of microbiome bacteria, that may be related to efficacy: for example, *R. faecis* was more likely to decline after treatment in non-responder oesophageal cancer patients, while increased in good responders. The monitoring of the variation of specific bacteria could be an alternative in evaluating response to treatments, since the faecal microbiota test is non-invasive and easy to be performed. However, these aspects need further investigations (90).

Recent research suggests that the interaction between host and gut microbiome could also affect the responsiveness to immunotherapies, likely due to a systemic activation of CD8+ Tcell stimulated by the release from gut bacteria of immunomodulatory molecules and metabolites. However, the mechanism is still unclear and under investigation (91). In particular, an important role of *Bacteroides* species in immunostimulatory modulation of CTLA-4 blockade has been identified, while *Akkermansia, Faecalibacterium, Clostridiale* and *Bifidobacterium spp* seem to be associated with PD-L1 inhibitors (88). Since resistance to immunotherapy is difficult to overcome, manipulating the gut microbiota could represent a promising strategy (92).

Based on this evidence, an emerging method for altering microbiota is the faecal microbiota transplantation (FMT), already used in some gastrointestinal disease as Inflammatory Bowel Disease (IBD), that allows to transplant stool information from healthy donors to patients (88). In a recent published clinical trial, long responder melanoma patients were used as donors of faecal stool for patient with a diagnosis of melanoma primarily resistant to immune checkpoint inhibitors: 6 patients out of 15 showed a radiological response, including objective response and stable disease. These data suggest that FMT could represent a method to overcome resistance to ICIs in melanoma (93).

For CRC, data are still immature. A pre-clinical trial on mice with MSS CRC showed that changes in the gut microbiome, due to the use of different antibiotics, affect the glycerophospholipid metabolic pathway and, consequently, the expression of immune-related cytokines: this results in a regulation of the therapeutic effect of immunotherapy in this subset of tumors, widely known for been resistant to ICIs (94).

In conclusion, data on the therapeutic aspects of microbiome are still immature and need further investigations, but, based on the important role that it plays in the regulation of the immune system, it seems to be a promising option for modulating response to immunotherapy.

## Author contributions

All authors made substantial contributions to this manuscript. AG, AP, SS contributed to the manuscript revision. AP and SS supervised the final version. All authors contributed to the article and approved the submitted version.
